# Postoperative Delirium, Learning, and Anesthetic Neurotoxicity: Some Perspectives and Directions

**DOI:** 10.3389/fneur.2018.00177

**Published:** 2018-03-20

**Authors:** W. Alan C. Mutch, Renée M. El-Gabalawy, M. Ruth Graham

**Affiliations:** ^1^Department of Anesthesia and Perioperative Medicine, University of Manitoba, Winnipeg, MB, Canada; ^2^Department of Clinical Health Psychology, University of Manitoba, Winnipeg, MB, Canada

**Keywords:** animal models, anesthetic neurotoxicity, biological scaling, postoperative delirium, postoperative cognitive dysfunction, surgical complications, non-human primate

## Abstract

Evidence of anesthetic neurotoxicity is unequivocal when studied in animal models. These findings have translated poorly to the clinical domain when equated to postoperative delirium (POD) in adults and postoperative cognitive dysfunction (POCD) in either children or the elderly. In this perspective, we examine various reasons for the differences between animal modeling of neurotoxicity and the clinical situation of POD and POCD and make suggestions as to potential directions for ongoing research. We hypothesize that the animal anesthetic neurotoxicity models are limited, in part, due to failed scaling correction of physiological time. We posit that important insights into POCD in children and adults may be gleaned from studies in adults examining alterations in perioperative management designed to limit POD. In this way, POD may be more useful as the proxy for POCD rather than neuronal dropout or behavioral abnormalities that have been used in animal models but which may not be proxies for the human condition. We argue that it is time to move beyond animal models of neurotoxicity to directly examine these problems in well-conducted clinical trials with comprehensive preoperative neuropsychometric and psychiatric testing, high fidelity intraoperative monitoring of physiological parameters during the anesthetic course and postoperative assessment of subthreshold and full classification of POD. In this manner, we can “model ourselves” to better understand these important and poorly understood conditions.

The United States Food and Drug Administration (FDA) safety communication regarding anesthetic and sedative agents for young children and women in their third trimester of pregnancy highlights the research leading to the concern for postoperative delirium (POD) and postoperative cognitive dysfunction (POCD) (https://www.fda.gov/Drugs/DrugSafety/ucm532356.htm). Research into these problems has become contradictory, and comparisons between small animal, non-human primate (NHP) and clinical trials have led to conflicting results, clouding recommendations for clinical management in both the pediatric and adult surgical populations. In this perspective, we will examine issues in animal modeling, differences in clinical studies, offer an instructive analogy with prior animal versus human research from another neurological condition, and offer suggestions that may help move the field forward.

## Overview

The preclinical experimental evidence is unequivocal that anesthetic agents [either *N*-methyl-d-aspartate (NMDA) receptor antagonists or gamma amino butyric acid (GABA) receptor agonists] are neurotoxic. These findings in combination with some, but not all, retrospective clinical reviews represent the transferrable knowledge forming the basis of the FDA communiqué. The recommendation cautions against lengthy or unnecessary surgeries for preschool children or for women late in their pregnancies because of the potential risk for early developmental damage to the child’s brain following anesthetic exposure (1, 2). However, three recent large retrospective-matched cohort studies involving almost 60,000 children, with control for sociodemographic and physical confounders, demonstrate no increased risk for cognitive impairment associated with exposure to one or even multiple anesthetics in children exposed from birth to 2 years of age (3–5). These findings contrast with a consistent body of work from the Mayo Clinic group, which shows an increased risk of learning disabilities in children following multiple but not single anesthetic exposure (6–9). Hansen has provided a comprehensive comparison of all but the most recent published literature and the strengths and weaknesses of the various clinical and animal studies relative to the pediatric population (10). At the other extreme of life, there is a well established association between older age and developing POD or POCD following operative interventions (11). However, an adult twin study showed no evidence of increased cognitive decline in the twin exposed to an operative intervention in later life (12) and a meta-analysis of adult studies has failed to indicate POD or POCD risk following anesthetic exposure in this population (13). What is the nature of these discrepancies between the preclinical findings of near-universal neurotoxicity and the contradictory or unsupportive clinical findings that have emerged as this research field starts to consolidate?

## Issues with Animal Modeling

The animal models used to investigate anesthetic neurotoxicity were based on an understanding of the critical role that NMDA- and GABA-mediated pathways play in normal neurodevelopment, coupled with the possibility that anesthetics with NMDA receptor antagonism might mimic the known detrimental effects of long-term ethanol and anticonvulsant exposure on these receptor subtypes (14). The animal models are maximized to assure measurable biomarkers to assess neuronal injury in exposed versus unexposed animals. In this regard, anesthetic agent exposure is often prolonged and administered in large and often outmoded dosage, such as high inspired concentrations of nitrous oxide, to establish a quantifiable lesion. A foundational principle is that the observable injury in the animal model correlates causally with either POD or POCD in the human condition, based on an assumption of a similar mechanistic arc for animal whole body and brain maturation—albeit on different time scales. In the developing brain, the period of maximal risk for exposure to anesthetic agents is believed to be the period of most vigorous synaptogenesis in the species under study (15, 16). In the rat pup, the most common experimental model of anesthetic neurotoxicity, this period is measured in days to weeks. In the NHP, the equivalent period occurs from the second trimester of pregnancy to 2 months of age. In the human neonate, maximal synaptogenesis occurs from the third trimester of pregnancy up to the first 2–3 years of life (17). Also important are issues raised by Hovens et al. (18) as to the differences between measurable lesions felt to represent markers of cognitive decline in experimental animals and the considerably more complicated modeling seen in humans.

### Scaling to Physiological Time

The discrepancies in the time course for biological processes between humans and animals as models for clinical disease have been highlighted recently by Agoston (19). He has shown time scales for a series of processes demonstrating the accelerated “pace of life” in the rat compared to the human. These temporal differences are from a minimum of 2.5 times faster in the rat for m/t RNA turnover to 84 times faster for sexual maturation. The direct translatability between two species is in large part predicated on interpretation of scale-free dimensionless modeling of biological mass or metabolism versus time. Thus, when plotting dimensionless mass versus dimensionless time a multitude of species can be shown to all fit the same hyperbolic growth curve (20). Under these conditions, this finding suggests experimental equivalence between species and provides credence to animal modeling to study the human condition. However, conditions of “experimental equivalence” are virtually never met and have not been properly considered in small animal modeling to study anesthetic neurotoxicity. Although scaling is usually considered for drug dosage in small animals due to their accelerated metabolism, and larger doses of drug on a mg/kg basis are administered, scaling for time is not corrected. As suggested by Agoston, this is a serious oversight. Physiologic time can be shown to scale to the body mass raised to the 1/4-power (M^1/4^) (21). To appropriately “model” anesthetic neurotoxicity with the rat pup serving as a surrogate for the human neonate, not only does the drug dose need to be scaled but so too does the time of exposure. Applying the power law scaling for time, the following can be derived: body mass of rat pup = 10 g; body mass human neonate = 2,500 g: ratio 250:1 which is the mass ratio now raised to the 1/4-power yielding a correction factor of 3.98. In the majority of rat pup experiments, an anesthetic exposure of 6 h is required to reproducibly result in clinically evident neurotoxic effect (22). Applying the power law scaling equivalence, the appropriate scaling for the human neonate would be an anesthetic exposure of 6 × 3.98 = 23.9 h. A neonate exposed to that duration of anesthesia would be essentially unprecedented although infants in the intensive care environment may be exposed to sedative agents for days to weeks. The calculation of time scaling can also be looked at inversely. For example, most anesthetic exposures in small children are limited to relatively short duration—typically 30–180 min for most common procedures. The rat pup equivalent exposure is then only 8–45 min—durations that have not been associated with any measurable signal of neuronal injury in the established animal models. Thus, when properly time-scaled, the limitations of small animals to model human anesthetic neurotoxicity become apparent and apply to the larger animal models as well. The use of NHPs has been suggested to more closely model the human neonate. The few studies reporting on postexposure cognitive function in NHP suggest that exposure time of 5 h is not associated with demonstrable deficits in behavior or memory (23) but 12–15 h are required for deficits to become manifest (7, 24). The birth weight of a rhesus monkey is approximately 500 g, yielding a ratio of 5:1 for the human infant. This ratio raised to the 1/4-power = 1.5, suggesting corrected exposure times for the human to be in the range of 18–22 h, consistent with the rat pup to human scaling corrections previously discussed. One argument advanced is that longer exposure in animal models may be equated to multiple anesthetic exposures in young children resulting in a risk of cognitive decline in the clinical situation. The recent series of large scale retrospective trials do not support this contention when examining children under 2 years of age exposed to up to four separate anesthetics (3, 4). Conversely, in support of evidence of anesthetic neurotoxicity is a recent study showing learning disabilities after multiple anesthetic exposure in a cohort of children, but not following single exposure (9).

### Limited Hemodynamic Monitoring

**Figure 1:**
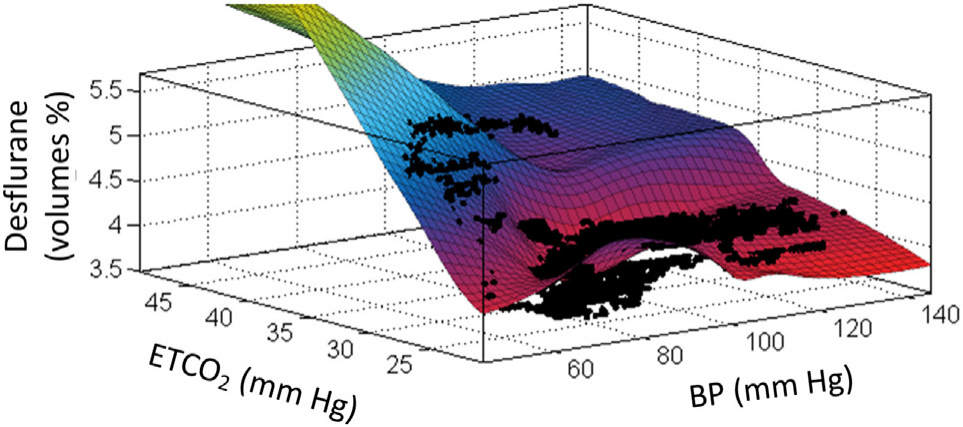
An example of the interaction between mean blood pressure (*x*-axis), end-tidal CO_2_ (*y*-axis), and end-tidal anesthetic agent (desflurane) concentration (*z*-axis) in one patient. This locally weighted smoothing linear regression had an *R*^2^ fit of 0.758 for these 13,014 data points. These data were collected intraoperatively in this patient using a data acquisition system downloading at 1 Hz. See the text and Ref. (25) for further discussion.

Hemodynamic and end-tidal gas monitoring are standard procedures for all clinically administered anesthetics. Equivalent monitoring in small animals is often difficult to achieve. The hemodynamic and gas exchange consequence of multiple hours of exposure in small animals to high doses of anesthetic agents is not usually considered. There are limited examples of single measures of arterial blood gases in rat pups at end experiment being equated to stability over the course of the experimental period. A single measure is not reflective of experimental stability, and the example cited indicated hypocapnic gas tensions that may be deleterious (22). More recent work suggests that intraoperative CO_2_ control may be an independent marker of POD risk (see below). Although NHPs offer a model in which hemodynamic and gas exchange may be monitored in a manner more similar to that used clinically, no studies to date report high fidelity intraoperative hemodynamic, ETCO_2_, or blood gas data to allow analysis over the course of the anesthetic exposure, although the monitoring is significantly improved over the earlier small animal studies. In many major centers, comprehensive intraoperative electronic monitoring is available clinically. Recently, high fidelity recording of the intraoperative anesthetic course has been examined in the clinical situation in the adult population. The inter-relationship between hemodynamic alterations, end-tidal gases, and anesthetic agent depth was examined *post hoc* looking for heretofore poorly examined interactions (Figure 1). In this manner, the intraoperative stress could be clearly delineated (25). Evidence is emerging that intraoperative delta CO_2_ may be a significant stressor for POD in susceptible individuals and tight control of end-tidal CO_2_ around the patient’s baseline normocapnic values may be an important modifier to limit POD in the adult (26). Although not yet studied in the pediatric population, this may also constitute a similar stressor for the developing brain whose immature vasculature is sensitive to both alterations in CO_2_ and perfusion (27).

## Analogy to Research into Subarachnoid Hemorrhage and Cerebral Vasospasm

Research into anesthetic neurotoxicity in many ways has followed a similar arc of progress for another neurological condition—subarachnoid hemorrhage and cerebral vasospasm. For this condition, increasingly complicated models were pursued from small animals to NHPs to large clinical trials (28, 29). Very promising interventions based on animal modeling consistently failed when applied clinically. Large clinical trials were required to refute the benefits of cerebral cooling and point the way for optimal timing for aneurysm clipping (30, 31). The way forward for management of this condition is increasingly by clinical trials in combination with sophisticated neuroimaging and big data analysis (32, 33).

## Limitations of Clinical Modeling of POD and POCD

The abovementioned limitations of animal modeling to define POD and POCD suggest that it is time to transition to direct clinical experimentation to further define these related problems (10, 34). Under debate is whether or not POD and POCD are separate entities (35, 36). If related can POD be a model to study POCD in humans? Hudetz et al. (37) showed a 14-fold increased in POCD following POD in cardiac surgery patients. Recently, common biomarkers for POD and POCD have been demonstrated (38). An enormous advantage of using POD as a proxy for POCD is immediate postoperative assessment instead of study over years. Prospective RCTs are significantly easier to design, model, and analyze for POD than for POCD, particularly in the adult population, but perioperative scoring systems for infants and children are being developed (39) or refined (40, 41). Pediatric POCD research to date has been largely limited to downstream assessment of cognitive performance after anesthetic exposure and is one of the reasons that this research clinically has largely focused on large-scale retrospective trials. However, it would be hugely advantageous if, for example, emergence agitation (EA) can be demonstrated to be a proxy for POD and POCD in children. There is evidence that there can be long-term consequences of EA (42, 43).

In adults, sequential study protocols can be designed to compare anesthetic agent administration, conduct of anesthesia, type of surgery for POD incidence, and duration as examples. Fruitful outcomes following these studies may then be used as models to apply to pediatric studies for comparison to longer term POCD outcomes. This comes with its own limitations but would at least allow for same-species comparisons.

**Figure 2:**
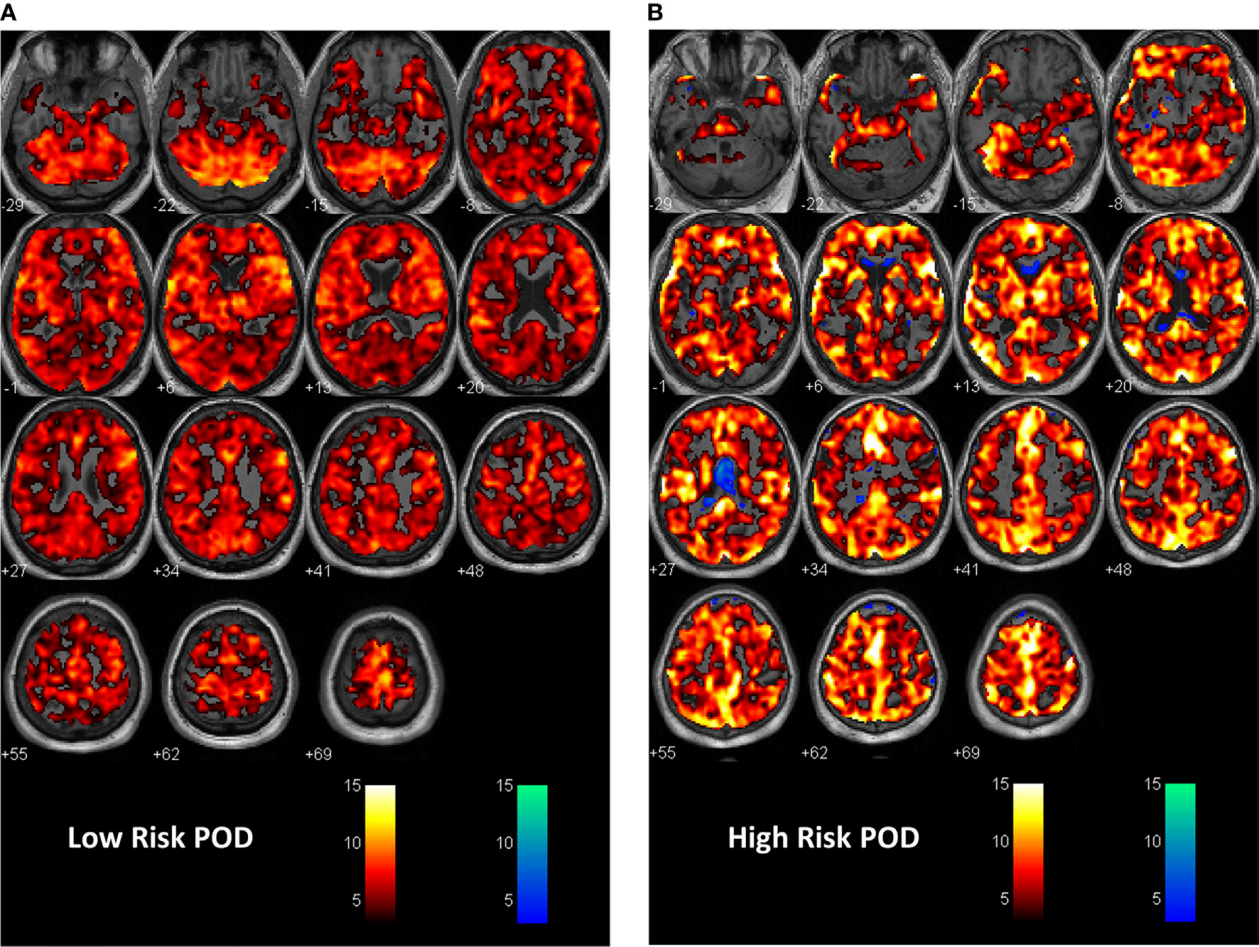
First level analysis with statistical parametric mapping (SPM) showing response to the CO_2_ stress test in **(A)** a patient at low risk for postoperative delirium (POD). In this instance, the expected response to the CO_2_ stimulus as recorded during BOLD imaging is shown. A vigorous response to CO_2_ is evident from the hot voxel response—shades of orange. The response at the *p* = 0.001 level occurred in 84% of whole brain parenchyma. The numbers below each image are the distance in millimeters above or below the anterior–posterior commissure. This patient had a non-POD outcome. The color bar is the *t*-value for fit to the general linear model from the SPM analysis. Voxels are colored if the *t*-value exceeded 3.11 in this instance. **(B)** A patient at risk of POD. Here, there is less response to the hypercapnic signal—a 64% response to hypercapnia and now an inverse or intracranial steal signal shown in cold voxels—shades of blue. The inverse voxel count was 4.3% of the total count. This patient had a subthreshold POD outcome. See Ref. (25) for a fuller description of the methodology.

Preoperative psychiatric and neuropsychological assessment tools have been increasingly utilized in carefully conducted prospective trials indicating the importance of the premorbid status of the patient for their risk of developing POD or POCD. Importantly, these comprehensive preoperative batteries do not have an animal equivalent. In some centers, advanced neuroimaging is shedding light on those patients at risk of POD. Most of this work relates to applied functional imaging to assess resting state network changes, alterations in the default mode network (44) and salience networks (45), and alterations in cerebrovascular reactivity to a CO_2_ stress test in patients deemed at risk (Figure 2). Work is also ongoing which assesses alterations in anatomic imaging in patients at risk compared to healthy controls. Computational programs are capable of delineating changes in regional tissue volumes or thickness that may clarify patients at cognitive risk. Brain imaging and the response to pain and alterations with the stress of anesthesia and surgery are also being entertained (34).

## The Stress–Diathesis Model of POD

**Figure 3:**
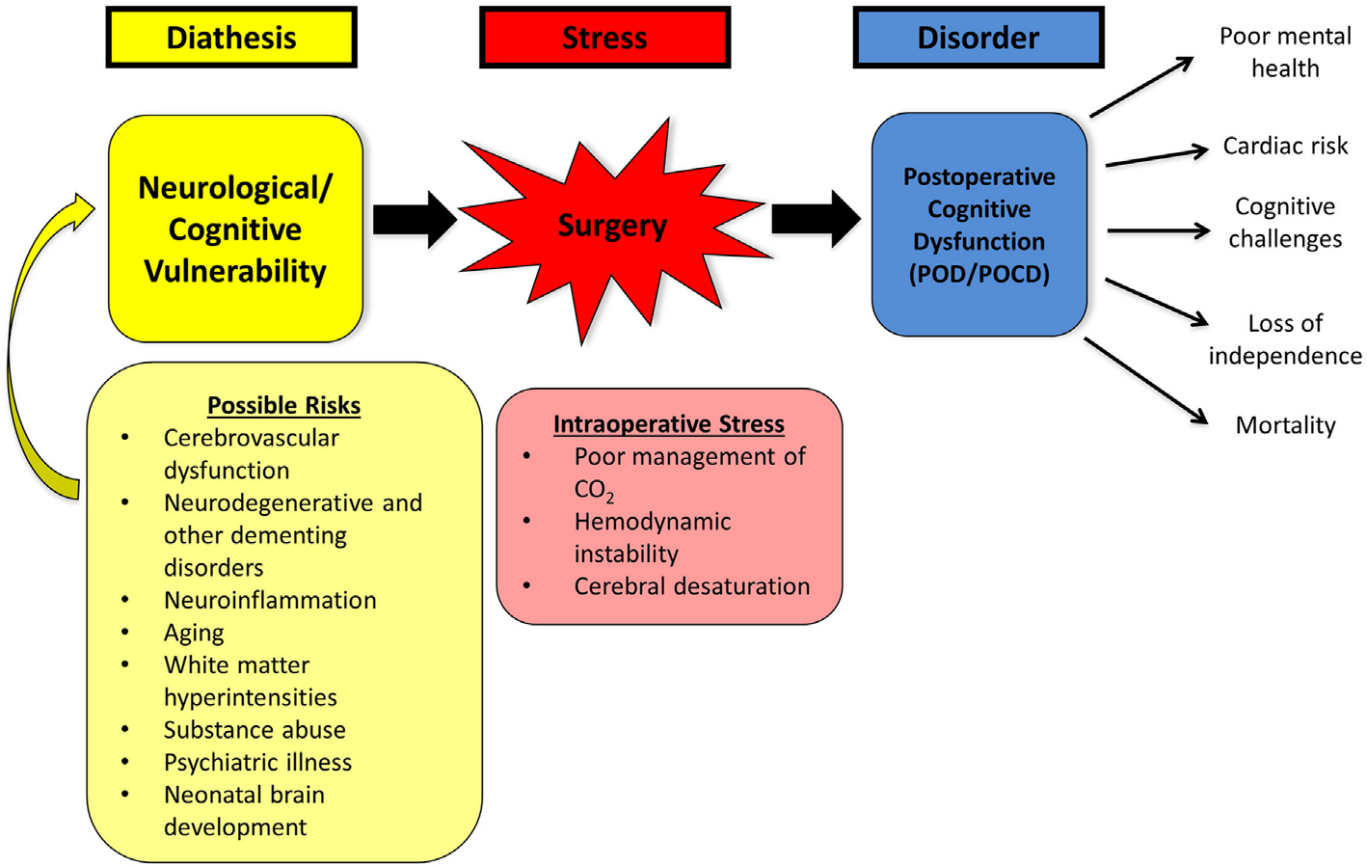
Diagrammatic depiction of the stress–diathesis model for postoperative delirium (POD). See the text for further details.

A stress–diathesis model of POD has been recently proposed (Figure 3) that moves away from the hypothesis that anesthetic toxicity is largely responsible for negative cognitive effects. In this model, the diatheses are the preoperative vulnerabilities that are delineated for an individual patient. These can include premorbid psychiatric history, drug dependencies, subclinical or clinical dementia, depression, and anxiety disorders as examples. The stress is the intraoperative anesthetic and surgical course. As noted earlier, high fidelity intraoperative monitoring provides insights not previously appreciated. The evolving model suggests three conditional states: (i) a very high risk group for POD with readily identified diathesis markers. This group is at risk irrespective of the anesthetic course, but potentially worsened by a poorly conducted anesthetic, (ii) a low risk group with no or few diathesis markers who do not manifest POD irrespective of anesthetic exposure or duration of procedure, and (iii) an intermediate group with evidence of premorbid diatheses placed at risk by an unstable anesthetic course. It is this third group where most gains to limit POD are likely to occur with better understanding of the stress–diathesis (25). As noted earlier, such a model can be adapted to the pediatric population based on the results from initial RCTs in adults.

## The Way Forward

To date, POD and POCD have been largely intractable problems. The health care costs of these conditions are immense (11, 46). The current working hypothesis is that anesthetic agents are an important contributing cause. The animal literature is strongly supportive of this premise, but the accumulating clinical findings would suggest other mechanisms or interactions not well delineated to date. The FDA drug safety communication has further stressed this situation. Suggestions offered in this perspective suggest other avenues for research study. The Noble Laureate Sydney Brenner realized that further understanding of genetic mechanisms required moving from unicellular organisms to study of the simple multicellular *Caenorhabditis elegans*. He also presciently stated the “the next model system is ourselves” (47). This suggestion may apply equally to our quest to understand POD and POCD in the pediatric and adult population.

## Author Contributions

WACM, RME-G, and MRG with conception, prior experimentation, writing, and final acceptance of manuscript.

## Conflict of Interest Statement

The authors have declared that no conflict of interest exists. The reviewer JW and handling editor declared their shared affiliation.
